# Supplementary material for: Testing for Causal Association between Serum Urate, Gout, and Prostatic Cancer in European Males
Source: medRxiv. 2025 May 11:2025.05.09.25327351. Preprint. [Version 1] doi: 10.1101/2025.05.09.25327351 (PMC12083634; doi:10.1101/2025.05.09.25327351)

**Supplementary Figure 1:** Forest plot of all non-hyperuricemia compartment of gout SNPs to prostate cancer

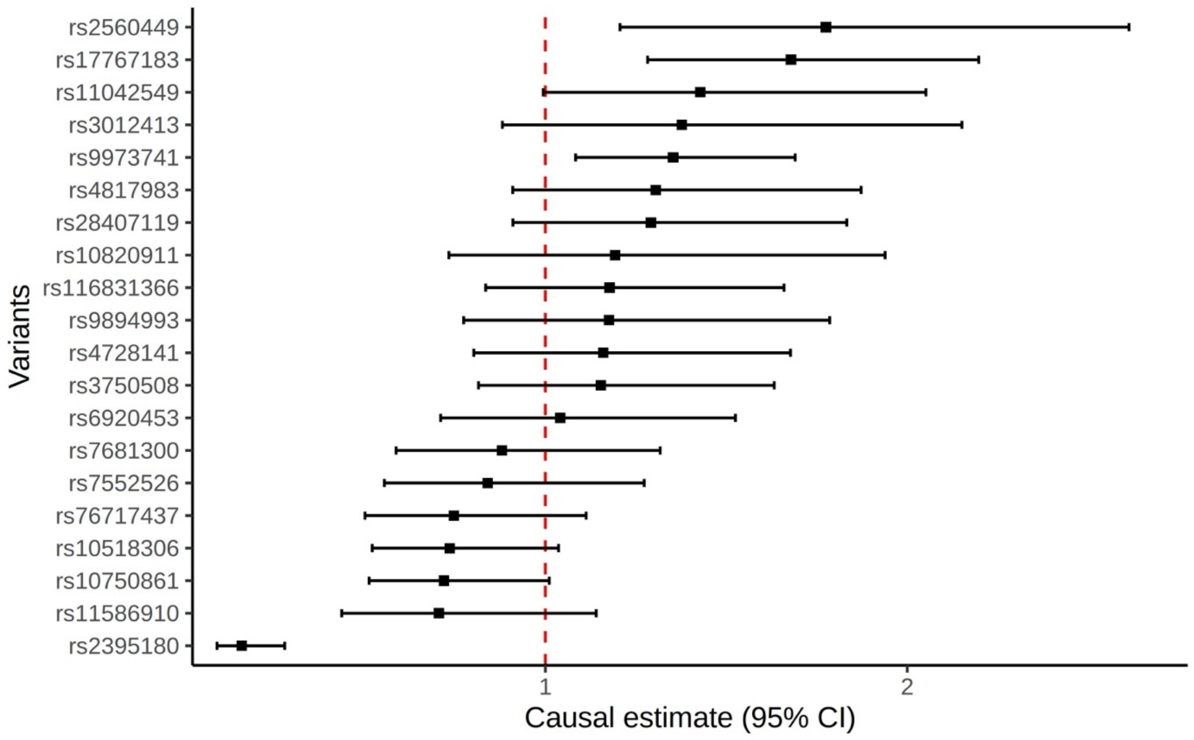

**Supplementary Figure 2:** LocusZoom plot of rs2560449 in prostate cancer (A) and in gout (B) in men.

A

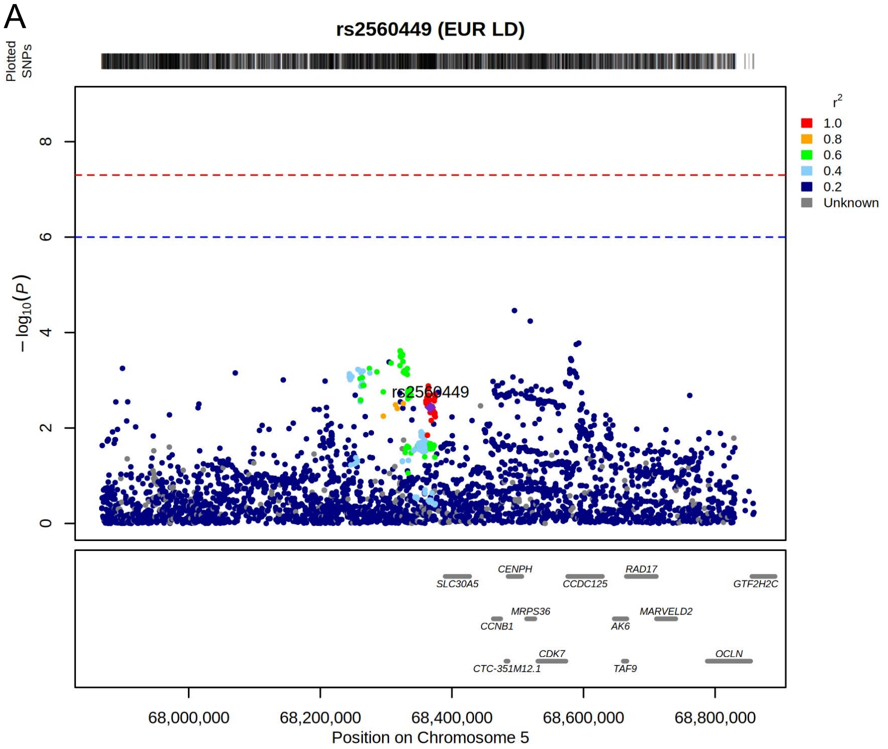


B

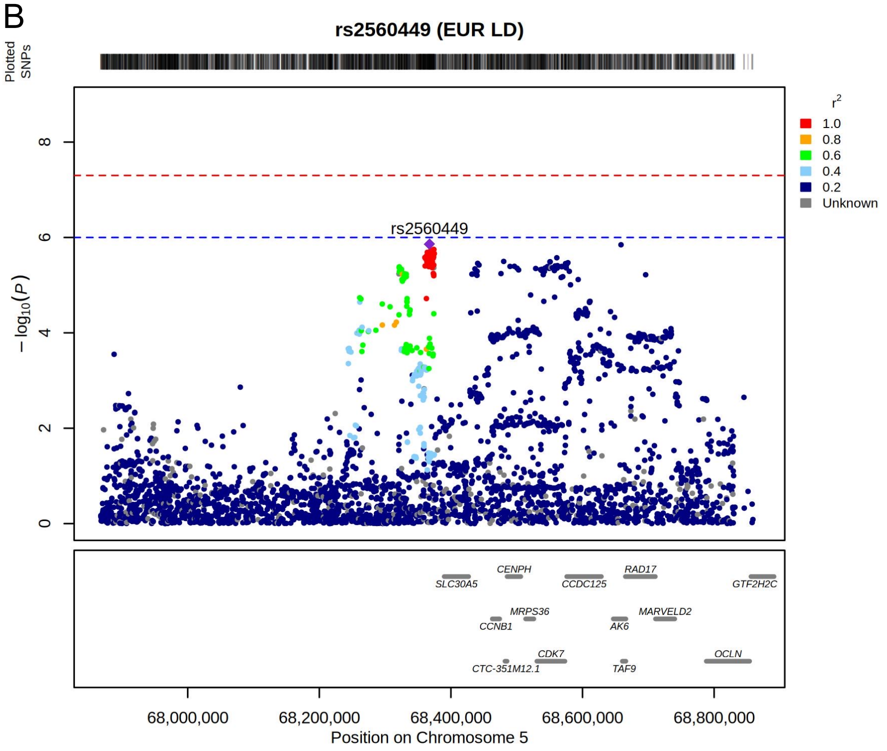

**Supplementary Figure 3:** LocusZoom plot of rs17767183 in prostate cancer (A) and in gout (B) in men.

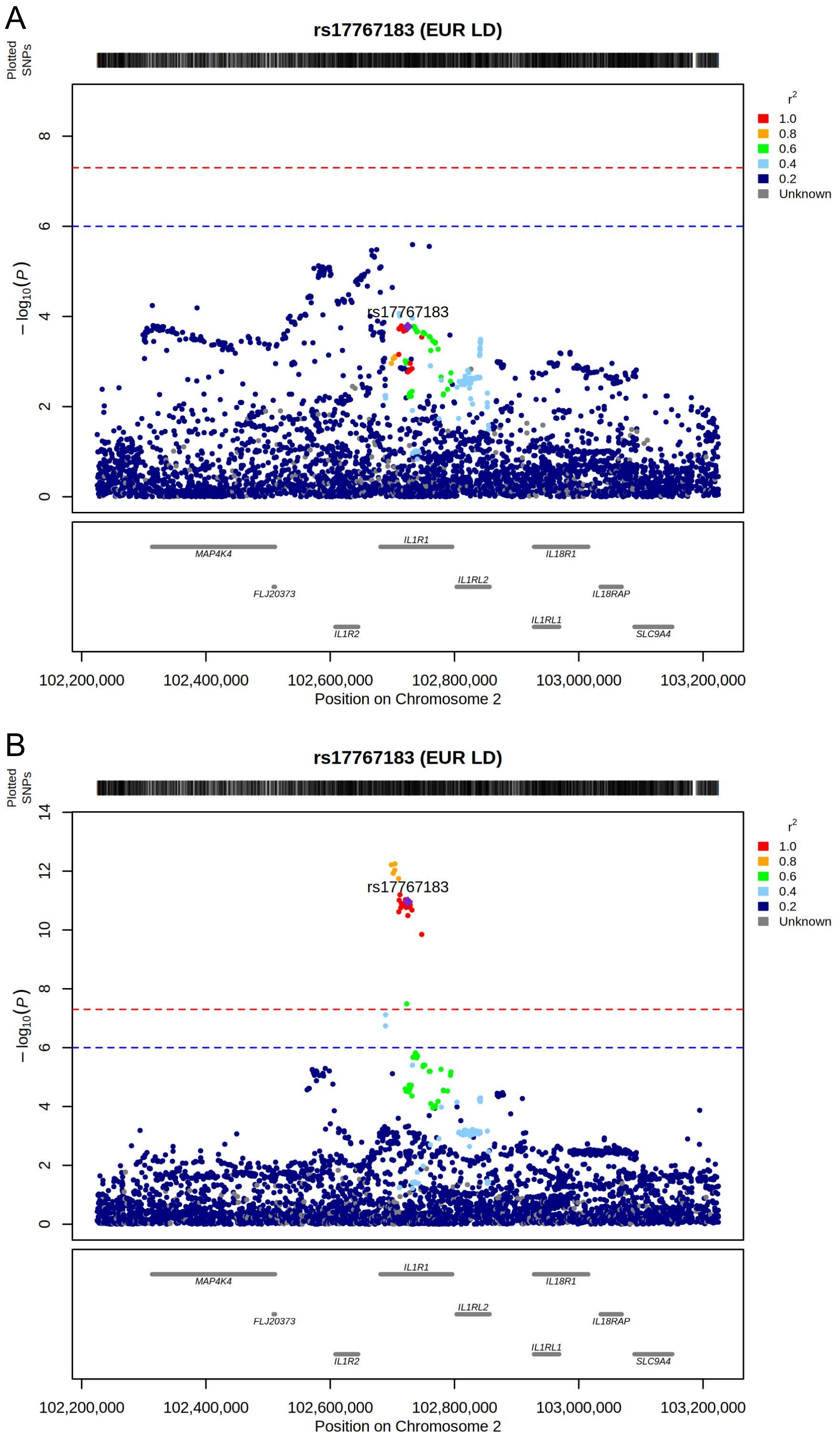

**Supplementary Figure 4:** LocusZoom plot of rs9973741 in prostate cancer (A) and in gout (B) in men.

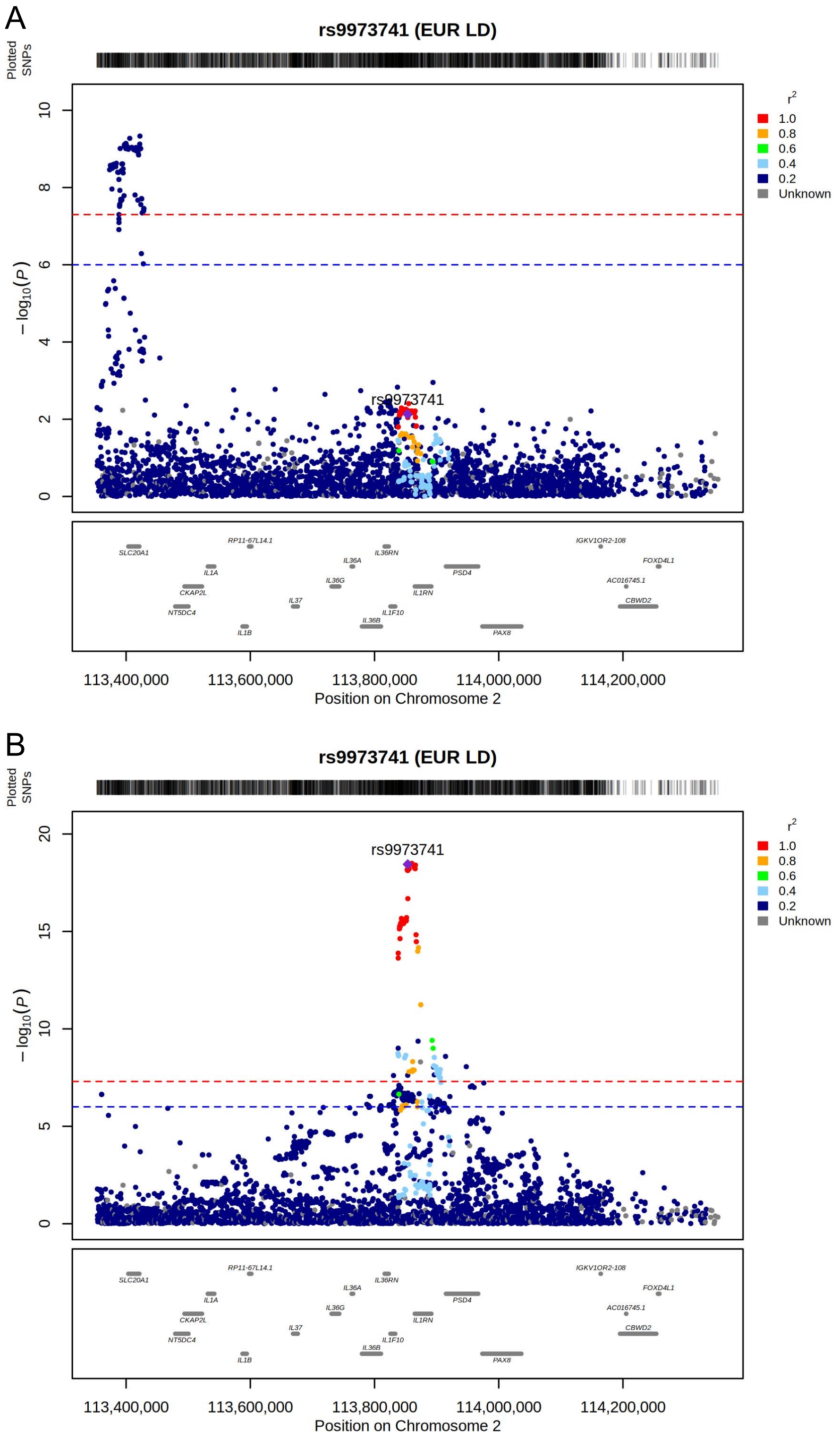

**Supplementary Figure 5:** LocusZoom plot of rs2395180 in prostate cancer (A) and in gout (B) in men.

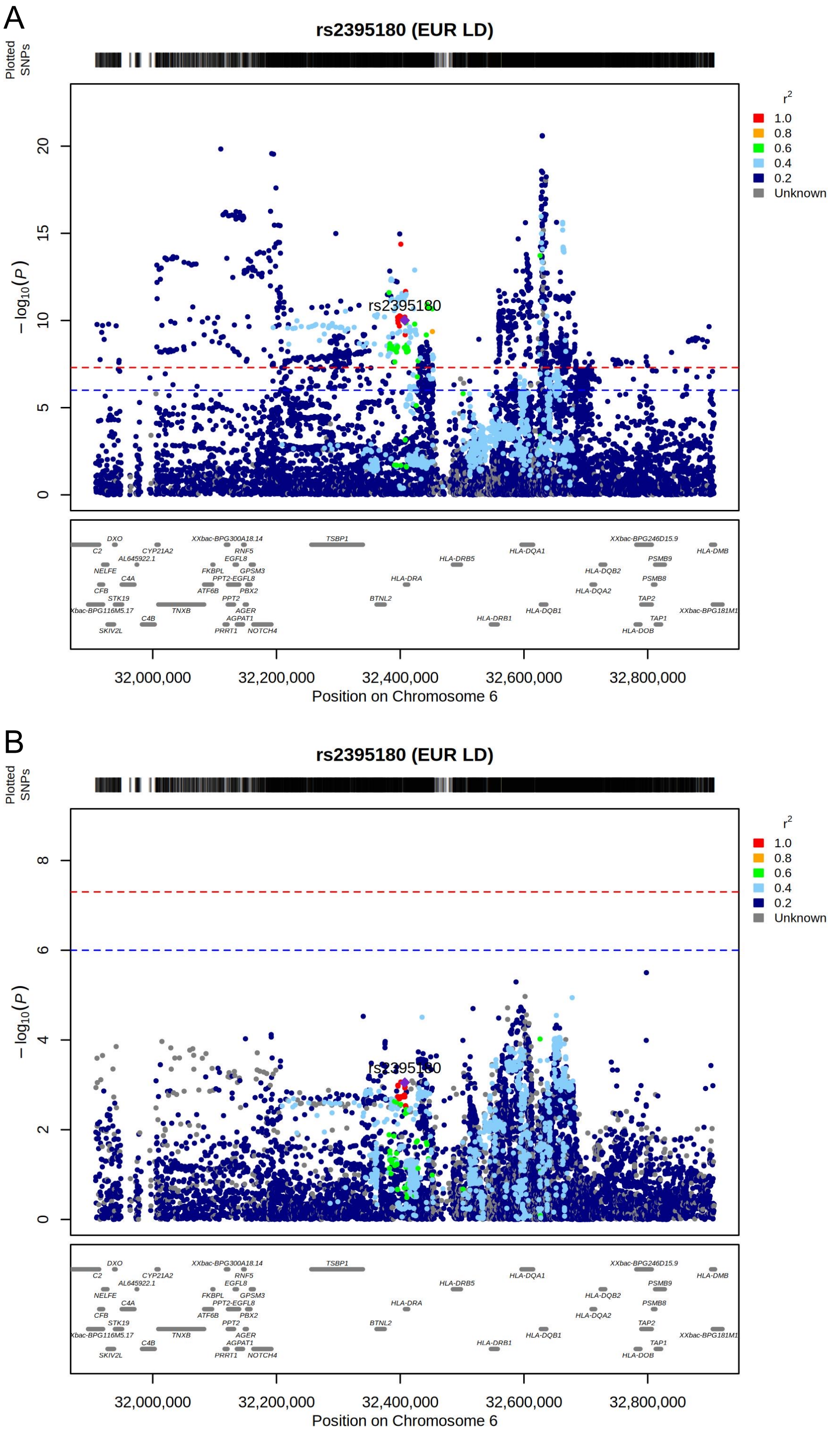

**Supplementary Figure 6:** LocusZoom of the eQTL for the association of rs9973741 and IL1RN in testis tissue (A) and with gout (B) and prostate cancer (C)

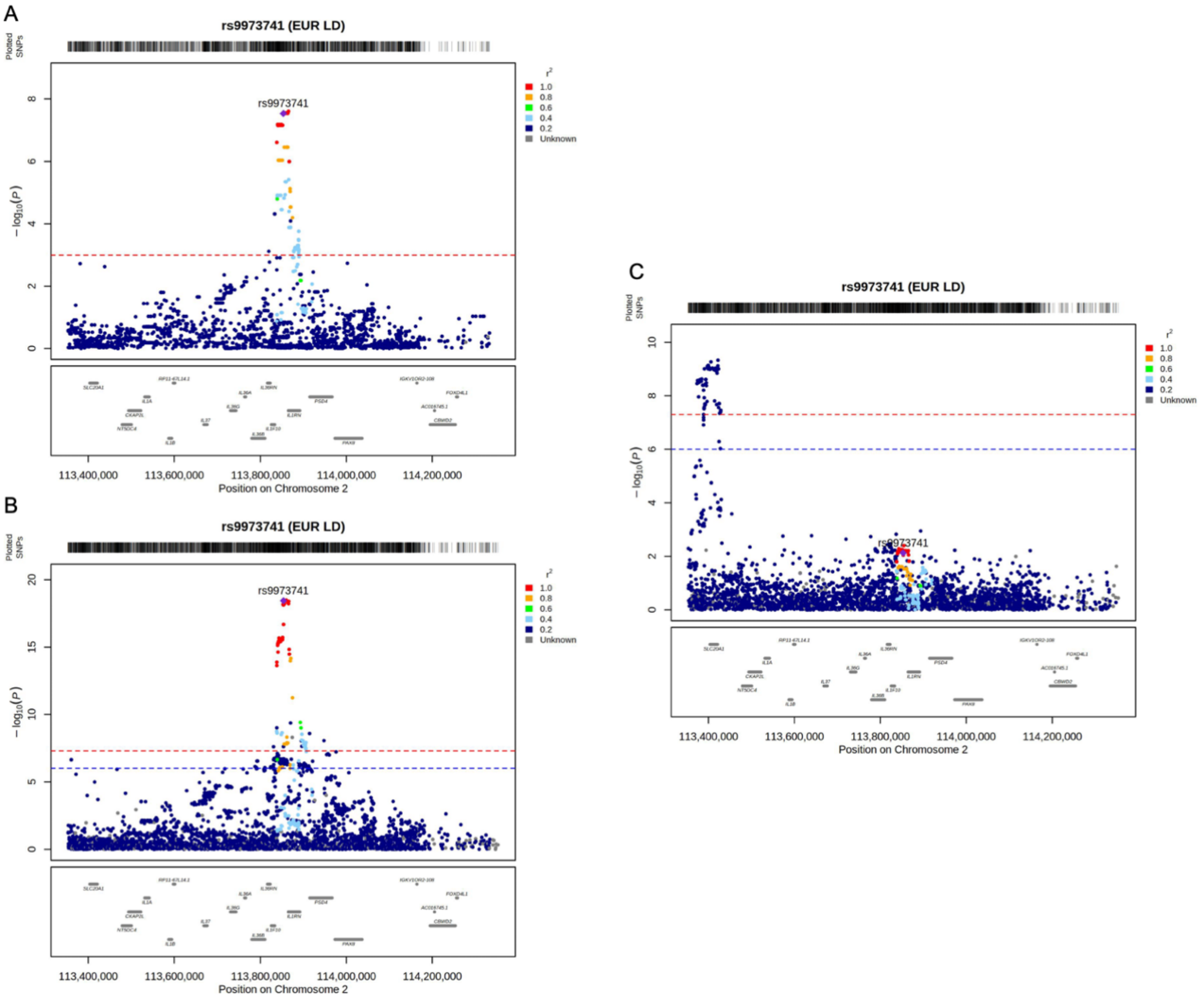

**Supplementary Figure 7:** Forest plot of all urate transporter SNPs to prostate cancer

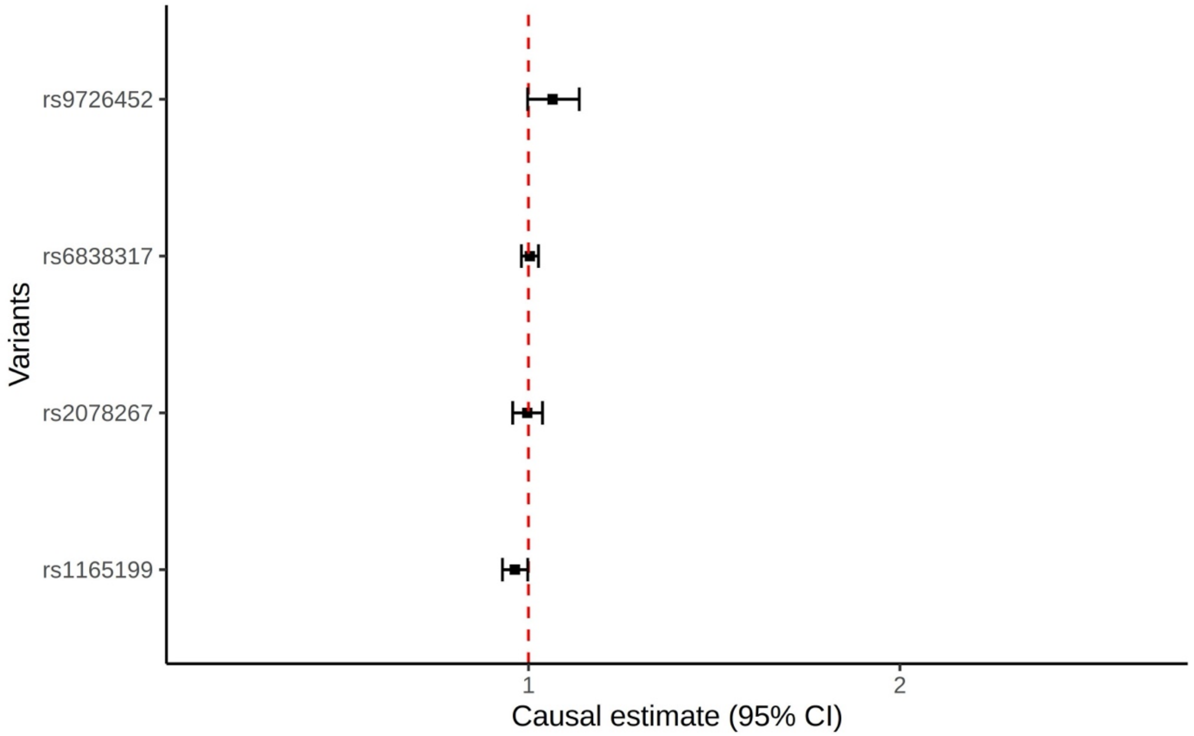

Supplement: Supplement 1 [file media-1.pdf]
